# Time for a proper career pathway for clinical trial managers?

**DOI:** 10.1186/s13063-023-07598-1

**Published:** 2023-09-01

**Authors:** E. J. Mitchell, E. Campbell, K. Goodman, J. Taylor, N. F. J. Youssouf, N. Wakefield, on behalf of the U. K. Trial Managers’ Network Executive Group, Claire Cochran, Sarah Cockayne, Riti Desai, Lucy Fletcher, Sarah A. Lawton, Ryonfa Lee, Kat Oatey, Joshua Savage

**Affiliations:** 1 Nottingham Clinical Trials Unit, Applied Health Research Building, University of Nottingham, University Park, Nottingham, NG7 2RD UK; 2 NMAHP Research Unit, Glasgow Caledonian University, Glasgow, G4 0NA UK; 3 Bristol Trials Centre, Population Health Sciences, Bristol Medical School, University of Bristol, 1-5 Whiteladies Road, Bristol, BS8 1NU UK; 4 Clinical Research Department, London School of Hygiene and Tropical Medicine, Keppel Street, London, WC1E 7HT UK

Clinical trial managers are vital to the design and delivery of clinical trials in the UK. They undertake a highly specialised, key role within the large, collaborative teams necessary to undertake high-quality clinical trials [1, 2]. Many trial managers are employed in Higher Education Institutions (HEIs), predominantly, though not always, in UKCRC-registered Clinical Trials Units (CTUs) [3]. As of June 2023, of 1393 UK Trial Managers’ Network (UKTMN) members, 80% (*n* = 1110) are employed by a HEI, of which 76% (*n* = 843) are based in a CTU. This article specifically focuses on trial managers employed by HEIs, rather than those employed by an NHS organisation. We refer to individuals in a trial management role as ‘trial managers’ throughout this article for brevity, and to summarise the profession generally, though acknowledge this includes a wide range of job titles, depending on local preferences and seniority.

Typically, in HEIs, trial managers are employed on one of two pathways or job families [3]. Though terminology differs between organisations, these can largely be described as “research” (also known as research/academic/research and teaching) or “professional services” (also known as administrative/professional/managerial). Whilst the core elements of a trial manager’s role remain the same irrespective of the pathway they are employed on, the two pathways are quite different in terms of recognition, professional development opportunities (including promotion), expectations/key indicators and other peoples’ perceptions.

Many staff in universities employed on a research pathway/job family often follow a similar trajectory, starting as a pre- or post-doctoral researcher and then potentially progressing to lecturer/assistant professor, senior lecturer/associate professor and professor roles. Key indicators for these roles typically involve leading research, by generating research income (i.e. grant awards) and high-impact publications and outputs. It is against these indicators that applications for promotion are usually assessed. Other job indicators and assessment criteria can include areas such as academic citizenship, at local, national or international levels, and wider engagement work. Whilst trial managers are indeed involved in many of these types of activities, they are often not leading their own research and thus not generating grant income. Some trial managers, despite playing a substantial role in the delivery of a clinical trial, are still unrecognised for their contribution and are not included in authorship teams, despite meeting ICJME criteria for authorship [4], a practice we strongly object to and have advocated for change [5]. In practice, this means that trial managers who are keen to progress their career, and employed on this job family/pathway, find it very challenging to demonstrate how they meet the criteria and are then overlooked for promotion. Furthermore, as their role is often funded via grant income, it can be particularly challenging to find protected time to progress in areas that do meet the demonstrative criteria.

By contrast, trial managers who are employed on a ‘professional services’ pathway/job family, typically do not have the option to progress their career via a promotion procedure, since this route usually only exists for researchers/academics in academia. Instead, they would need to demonstrate that their *role* has changed such that the role requires re-grading, rather than focussing on the individual and their development. Of course, irrespective of which pathway an individual is employed on, trial managers also have the option of applying in open competition for a vacant/new position at a higher level, should one exist.

Not all trial managers are interested in ongoing progression and promotion, nor do we suggest they should be. However, we do firmly believe that there should be a career pathway in place for trial managers to progress their careers *if* they want to. In a previous survey of 433 UKTMN members, 50% of participants reported never having progressed to a more senior role, a third of which was due to “no career pathway in my organisation” [3]. Furthermore, having an ‘unclear career pathway’ was in the top 3 barriers to career development reported. We recognise there is always a ‘ceiling’ for any profession — in academic clinical trials this may stop at roles such as Professor or Director/Head of Operations. In many universities, however, more experienced, and senior trial managers are unable to progress to these roles, either because they cannot demonstrate they meet the indicative criteria (for academic posts), or because more senior roles simply don’t exist. UKTMN members have reported for many years that this is a problem and, as outlined in their Professional Development Strategy [6], UKTMN are actively advocating for change.

For some time, there have been murmurings of a ‘third career pathway’ — essentially, an alternative career pathway/job family that more accurately reflects the specialist and technical nature of this type of role. This could be applicable to many roles within universities, not just trial managers, for example, people who identify as a methodologist [7] or the vast amount of people who are in key specialist technical roles and are crucial to research and development in the UK. This concept is not a new one: in their 2016 report, the Academy of Medical Sciences provided ten key recommendations for improving the recognition of team science contributors [8]. Recommendation 10 focussed on the need to provide clear career paths and development opportunities. Clinical trials are multidisciplinary and encapsulate the essence of team science — all who contribute to their design and delivery should be recognised, not simply the lead investigator.

The idea of exploring a ‘third pathway’ should be done with caution. How best to provide trial managers with a clear career pathway is a complex problem and it would be foolish to think otherwise. Understanding the needs of trial management roles and wider clinical trials is a critical element to addressing this complex issue.

To gain a deeper understanding, two national online surveys were undertaken. Trial managers (UKTMN members) were asked about the career pathway they are currently employed on, whether they were satisfied with this pathway, and the advantages and disadvantages they perceived of being employed on this pathway. Three hundred and twenty-four out of 892 (36%) responses were received from the membership. In addition, a separate survey was sent to 52 UKCRC-registered Clinical Trials Units, to understand what career pathways trial managers (of different levels of seniority) were employed on, the reasons for selecting this pathway and whether they had either investigated or implemented an alternative pathway. Thirty-seven (71%) responses were received. The full results of both surveys can be found here: https://www.tmn.ac.uk/resources/uktmn-job-family-survey.

Survey data and subsequent discussions with UKTMN members at an in-person event at the International Clinical Trials Methodology Conference (ICTMC) in October 2022 have clearly demonstrated that a one-size fits all approach is not appropriate. There are advantages and disadvantages to both currently available pathways, from a trial manager and CTU perspective. Forty per cent of trial managers reported dissatisfaction with the pathway they are currently employed on: 18% reported they would prefer to be employed on the other pathway (i.e. if they were on a research pathway they’d rather be on the professional services pathway and vice versa) and 22% reporting that neither of the currently available pathways was suitable. Interestingly, of the sample of trial managers who responded, many were currently employed in a more senior role, confirming our long-held view that the issue of lack of career pathway is even more pertinent to more senior roles, as they hit the glass ceiling with no prospect of progression, in their current profession, in the future. Over half of the respondents (181/324, 56%) were employed on the Professional Services pathway, with around a third on the Research pathway (104/324, 32%) and other respondents either not knowing which pathway they were employed on (31/324, 10%) or being employed on another pathway (8/324, 3%), e.g. ‘academic-related’ and ‘research and professional services’. There were a huge variety of opinions on the perceived advantages and disadvantages of the currently available pathways. Trial managers on the professional services pathway reported that they appreciated there was no expectation to produce academic outputs, nor be judged against a promotion framework that has been developed for academics. However, others felt the significant input they give into a trial is not recognised nor given the same level of merit as academic colleagues, and that this could be improved if employed on an alternative pathway. With respect to career progression, some reported that clear structures were in place, whereas others reported a lack of development opportunities and no roles, retaining their specialism, at higher levels in the organisation. Similarly, for trial managers employed on a research pathway, there was an overlap between perceived advantages and disadvantages. Some reported that the research pathway enabled them to apply for promotion, unlike their professional services colleagues, though many recognised their role did not naturally fit the typical academic pathway, restricting progress. With respect to recognition, some perceived that the research pathway is more well respected in the university setting, recognising the intellectual input the trial manager provides, whilst others reported that, at university level, staff had difficulty in understanding the different nature of the role. Although many trial managers relish the opportunity to get involved in activities outside of the day-to-day running of their trial [3], some felt that being employed on the research pathway was a hindrance, since there was a higher expectation to get involved in developing their own research portfolio, which was challenging due to time constraints within their busy trial manager role. CTUs reported a blend of both pathways being used, with more senior staff tending to be employed on the research pathway. The reasons for CTUs selecting the career pathway they had, broadly fell into four categories: (i) it was a university requirement/precedence of previous similar posts, (ii) to enable career progression, (iii) the pathway fulfilled the requirements of the role of trial manager, (iii) financial considerations, e.g. posts under the research pathway attracting institutional indirect costs in grant costings, unlike professional services posts. Interestingly, some CTUs reported that trial managers could be hired on either pathway, depending upon the individual’s career aspirations, and that appointing on a professional services pathway enabled switching to an academic pathway at a later date, if desired. Nine CTUs (9/37, 24%) reported that an alternative pathway was either being investigated *(n* = 6), implemented (*n* = 1) or has been implemented (*n* = 2).

To our knowledge, the University of Liverpool is the first UK university to recently launch an alternative pathway that could be appropriate for trial managers. The Research Technical Professional (RTP) career pathway has been developed for people who have developed specialist skills, whether that is from a research, technical or management perspective, but who are not academics nor lead investigators. Before implementation, the University of Liverpool consulted widely, across all faculties and many job roles, ensuring wide stakeholder engagement. The grading structure within the RTP pathway enables individuals to ‘opt-in’ (i.e. it is not mandatory) to the pathway and progress to the highest grade within the university (equivalent of professor), if desired, with indicators appropriate to these types of roles. The RTP terminology aligns with the language used in the UKRI’s commitment to the Technician’s commitment [9] to this group of staff, again with a broad definition being applied, and of high relevance to trial managers. This new pathway could be a way in which trial managers are recognised for the specialist nature of their role enabling career progression, without them having to take a sideways step to either a solely academic role or a senior professional services role (unrelated to their specialism), enabling them to maintain their specialist skills.

This is, of course, just one way of addressing this problem. Alternative solutions could be having broader promotion criteria and key indicators for both of the current pathways, enabling them to be applicable to a wider range of staff and roles, implementation of a promotions procedure for professional services staff, or offering individuals the opportunity to ‘switch’ between pathways, depending upon their skills, experience and development plans for the future, as some CTUs have already implemented.

Regardless of the pathway that trial managers are employed on it is crucial they are recognised for the specialist skills they hold, and opportunities are created to ensure their career development. This includes ensuring trial managers are named on publications, recognising the significant contribution they have made to a clinical trial, supporting requests for training and development and, in the simplest of terms, not judging applications for promotion by criteria that are simply not suited to the job role.

A sector-wide change is urgently needed. Progress is being made, but it is slow, and, in the meantime, the trial management profession is at risk of losing individuals with significant expertise, because they are unable to progress their careers currently in HEIs [10]. It is the responsibility of us all, to advocate for change. Trial Managers should hold discussions within their local departments. Departments/CTUs should engage in discussions within their university schools and faculties, engaging with change agents elsewhere in the organisation. Universities that have made progress in this area should share their experiences for others to learn from. Many universities are signatories to the Technician’s Commitment (https://www.techniciancommitment.org.uk/), which “aims to ensure visibility, recognition, career development and sustainability for technicians working in higher education and research across all disciplines”, and have committed to submitting action plans outlining their commitment to this initiative. Whilst we may not consider trial managers to be ‘technicians’ per se, many of the structural barriers to professional development are consistent with those faced by technicians, which the Technician’s Commitment was established to break down. In their development plans to demonstrate their commitment to the Technician’s Commitment, universities should ensure the wide range of specialist roles, including trial managers, have appropriate career pathways in place to ensure the sustainability of the profession in the future.

In summary, trial managers should be afforded the same development opportunities as others, crucially by having an established career pathway, suitable to their role, in order to ensure we retain the skills and expertise needed to manage clinical trials.

## Data Availability

Not applicable.

## Abbreviations

CTU: Clinical Trials Unit
HEI: Higher Education Institution
UKRI: UK Research and Innovation
UKTMN: UK Trial Managers’ Network
NHS: National Health Service
ICJME: International Committee of Journal Medical Editors
ICTMC: International Clinical Trials Methodology Conference
